# The prevalence of the Val66Met polymorphism in musicians: Possible evidence for compensatory neuroplasticity from a pilot study

**DOI:** 10.1371/journal.pone.0245107

**Published:** 2021-06-09

**Authors:** Tara L. Henechowicz, Joyce L. Chen, Leonardo G. Cohen, Michael H. Thaut

**Affiliations:** 1 Music and Health Sciences Research Collaboratory, Faculty of Music, University of Toronto, Toronto, Canada; 2 Faculty of Kinesiology, University of Toronto, Toronto, Canada; 3 National Institute of Health/National Institute of Neurological Disorders and Stroke, Bethesda, MD, United States of America; 4 Faculty of Medicine, Institute of Medical Sciences, University of Toronto, Toronto, Canada; National Institutes of Health, UNITED STATES

## Abstract

The study compared the prevalence of the Val66Met Brain-derived Neurotrophic Factor single nucleotide polymorphism (rs6265) in a sample of musicians (N = 50) to an ethnically matched general population sample from the 1000 Human Genome Project (N = 424). Met-carriers of the polymorphism (Val/Met and Met/Met genotypes) are typically present in 25–30% of the general population and have associated deficits in motor learning and plasticity. Many studies have assessed the benefits of long-term music training for neuroplasticity and motor learning. This study takes a unique genetic approach investigating if the prevalence of the Val66Met BDNF polymorphism, which negatively affects motor learning, is significantly different in musicians from the general population. Our genotype and allele frequency analyses revealed that the distribution of the Val66Met polymorphism was not significantly different in musicians versus the general population (p = 0.6447 for genotype analysis and p = 0.8513 allele analysis). In the Musician sample (N = 50), the prevalence of the Val/Met genotype was 40% and the prevalence of the Met/Met genotype was 2%. In the 1000 Human Genome Project subset (N = 424), the prevalence of Val/Met was 33.25% and the Met/Met genotype prevalence was 4%. Therefore, musicians do exist with the Val66Met polymorphism and the characteristics of long-term music training may compensate for genetic predisposition to motor learning deficits. Since the polymorphism has significant implications for stroke rehabilitation, future studies may consider the implications of the polymorphism in music-based interventions such as Neurologic Music Therapy.

## Introduction

Musicians serve as excellent models for studying neuroplasticity of the sensorimotor system. Music training uniquely involves long-term highly specific motor learning, often begins early in age, and involves error learning and multisensory feedback [1, 2]. However, genetic differences may influence both the likelihood of becoming a musician and the effects of music-induced plasticity [3]. The Val66Met Brain-derived Neurotrophic Factor single nucleotide polymorphism (rs6265) (Val66Met BDNF SNP) is a common mutation present in 25–30% of the general population [4] that is associated with possible deficits in motor learning and neuroplasticity [5–7]. Met-carriers show decreased activity-dependent secretion of pro-Brain-derived Neurotrophic Factor (pro-BDNF), which alters the secretion of mature-BDNF, NMDA-receptor long-term potentiation (LTP), long-term depression (LTD), and the formation of inhibitory synapses [8]. Due to the role of pro-BDNF in LTP processes, BDNF is a critical protein for learning, neuroplasticity, and rehabilitation [9].

In healthy populations, Met-carriers’ (Val/Met and Met/Met) motor learning deficits can be described by differences in error learning [6], short-term plasticity and cortical-excitability of M1 [5, 7, 10], and interhemispheric transfer of motor skills [11, 12]. Kleim et al. (2006) found that Met-carriers compared to Val/Val homozygotes showed decreased activity-dependent short-term plasticity (measured by motor-evoked potentials) in M1 following 30 minutes of first dorsal interosseous muscle exercise [13]. However, after intense training (12 days of marble navigation training of the first dorsal interosseous muscle) healthy Met-carriers can overcome deficits in short-term plasticity and do not show differences in long-term cortical-motor map plasticity [10]. Met-carriers also show deficits in motor learning with Transcranial Direct-Current Stimulation (tDCS) applied to the motor cortex (M1), where motor learning is usually enhanced by anodal tDCS. Since Met-carriers have decreased activity-dependent BDNF, tDCS does not enhance motor learning or corticospinal excitability in Met-carriers [7]. Although there is a variety in stimulation protocols used to examine differences between Val/Val and Met-carriers [for a review, see 5], there is evidence to suggest that the presence of the Met-allele (of the Val66Met polymorphism) decreases healthy participants’ responses to stimulation protocols and activity-dependent short-term plasticity.

Deficits of Met-carriers also include limited stability of white matter structural connectivity [14], interhemispheric transfer of a motor skill [11], visuomotor adaptation [6], and complex motor skill learning [15, 16]. Juondi et al.’s (2012) study compared participants with the Val/Val genotype to the Val/Met genotype on motor performance and rate of learning in a visuomotor task during the learning period, after 45-minutes retention, 24-hour retention, and at 8-months for de-adaptation. Met-carriers showed deficits in learning and 24-hour retention and larger deficits with larger perturbations [6]. With more complex tasks such as a backhand baseball pitch, Met-carriers compared to Val/Val genotypes showed deficits in 48-hour retention and showed greater error in distance from the target [15]. In a study examining early- and late- periods of motor skill learning of a basketball shooting exercise, Met-carriers compared to Val/Val showed different sensory-motor integration patterns which may be associated with poorer learning of the skill [16]. These profound deficits in learning and adaptation provide possible evidence for irregularities in cortico-cerebellar motor system function, which is implicated in the early phases of motor learning [17].

Conversely, musicians have enhanced motor and sensory skills and earlier onset of music training is associated with greater enhancements in sensorimotor learning [18, 19]. Long-term music training is a catalyst for neuroplasticity as musicians show numerous structural brain adaptations, functional changes in auditory-motor and sensorimotor networks, and white matter tract and corpus callosum integrity [for reviews see 1, 2]. These structural adaptations are more pronounced in early-trained msusicians, such as greater reorganization of the primary motor cortex (measured by intrasulcal length of the precentral gyrus) [20]. After paired associative stimulation (PAS) musicians compared to non-muscians show enhanced LTP and LTD mechanisms with steeper motor evoked potentials and short-latency intracortical inhibition [21]. Recent evidence from a neurophysiological study of pianists versus non-musicians revealed that neural circuits for tactile-motor and proprioceptive-motor integration functions are reorganized in pianists, enabling fast and dexterous finger movements and rapid adjustment of movements [22]. Together, musicians’ long-term motor training and specialization leads to structural, functional, and neurophysiological changes that likely require intact plasticity mechanisms such as BDNF-dependent LTP.

Behaviourally, musicians compared to non-musicians have enhanced audiomotor synchronization, faster reaction times on sensory and multisensory tasks, better performance and learning of tasks that require fine motor skills, and superior interhemispheric transfer. Musicians’ enriched audiomotor synchronization and error correction mechanisms are represented by decreased variability and better accuracy than non-musicians in tapping tasks when coordinating actions with external auditory cues [23–25]. Musicians outperform non-musicians with faster reaction times during spatial [26] and multisensory integration tasks [27]. Musicians showed greater accuracy on the motor sequence task, a repitition task of learned sequence key presses, than non-musicians during the training session and music experience was related to better performance on retention following both 12-hours of sleep or awake conditions [28]. In another study, early-trained musicians (before age of 7) outperform later-trained musicians on a timed motor sequence task [19]. Long-term training may benefit motor skill learning as musicians compared to non-musicians have greater accuracy at imitating actions after waiting videos of hand gestures, with greatest accuracy for fine motor finger movements [29]. Consistent with findings of corpus callosum integrity in musicains [30], music training improves interhemispheric transfer and communication, where musicians show greater accuracy than nonmusicians on the fingertip cross-localization test [31]. In a practical example of skill acquisition, participants with piano expertise or no expertise learned to complete surgical knots and procedures. Pianists compared to non-musicians received higher scores on the standardized rating system, the Objective Structures Assessment of Technical Skills [32].

Since the Val66Met polymorphism is associated with deficits in motor learning and activity-dependent plasticity, the Val66Met polymorphism is a great candidate gene for investigating the relationships between music training and cortical plasticity. Based on the behavioral evidence of enhanced motor performance capabilities and the physiologic evidence of associated neural plasticity in musicians, we therefore predict a significantly reduced prevalence of the Val/Met genotype polymorphism in musicians when compared to the general populations.

To test this prediction, the objective of this pilot study is to investigate the prevalence of the Val66Met BDNF single nucleotide polymorphism (SNP), a genetic mutation associated with deficits in neuroplasticity and motor learning, in a sample of musicians (N = 50) compared to the general population (N = 424) subset from the 1000 Human Genome Project.

## Methods and materials

Ethical approval was obtained from the University of Toronto Research Ethics Board; all participants provided written informed consent. For the control sample, genotype data were extracted from N = 424 European samples from the 1000 Human Genomes Project (HGP). The 1000 HGP has genotype data on 2318 individuals from 19 populations in 5 continental groups, generated on the Illumina Omni2.5 platform. We performed extensive quality control analyses and extracted a set of 1752 unrelated samples with high genotype quality. The subset included 119 Utah Residents with Northern and Western European Ancestry (CEU) samples, 110 Tuscan in Italy (TSI) samples, 95 GBR (British in England and Scotland) samples, and 100 Iberian in Spain (IBS) samples. We did not have demographic information for the 1000 HGP subset. For the control dataset, genotype data was used to infer sex for each individual.

We recruited a cohort of N = 50 healthy musicians, currently enrolled in or recently completed a bachelor’s degree in music performance (within 5 years) with four grandparents of descent from a European country (excluding Finland) matched to the 1000 HGP subset. We recruited European ancestries to control for the variation in the Val66Met prevalence between ethnicities [4]. We recruited an equal number of males and females. For the musician sample, we recorded demographic variables of age, sex, degree program and year, primary instrument, years of primary instrument training, secondary instruments, and special musical achievements.

### Genotyping

Musicians provided a saliva sample using the DNA Genotek OG-500 kits. Genotyping for the SNP rs6265 (BDNF; Val66Met) was performed at The Centre for Applied Genomics, The Hospital for Sick Children, Toronto, Canada using a pre-designed TaqMan® SNP Genotyping Assay (C__11592758_10, Life Technologies Inc., Carlsbad, CA, USA). The 10 ml reaction mix consisted of 5ml TaqMan Genotyping Master Mix (Life Technologies), 0.25 ml of 40X combined primer and probe mix, 2.75 ml water and 20–50 ng of DNA template. Cycling conditions for the reaction were 95°C for 10 min, followed by 40 cycles of 94°C for 15 sec and 60°C for 1 min. Samples were analyzed using the ViiA™ 7 Real-Time PCR System and analyzed using ViiA™7 software.

### Statistical analysis

Statistical analyses were conducted in R. Due to the small cell count (<5), Fisher’s exact test was used to assess significant differences in genotype frequencies and the Chi-square test was used to detect significant differences in allele frequencies (two-tailed and alpha = 0.05). Due to the small count for the Met/Met genotype (N = 1), we compared the demographic variables between Val/Val and Met-carriers (Val/Met and Met/Met). We conducted two-sample t-tests for age, total years of training, and years of training on the primary instrument. We did not stratify by instrument type due to sample size.

## Results

The mean age of the musician sample was 21.8± 3.5 years with 11.7± 4.7 years of training on their primary instrument and 14.3± 3.6 years of total music training (See Table 1). The musicians included instrumentalists, woodwind (N = 16), brass (N = 7), strings (N = 15), and percussion (N = 4) as well as keyboard players (N = 8). Voice majors were not included because singing may involve different neural processes from instrumental music training, where motor learning involving the upper limbs are critical to instrumental music performance. N = 37 musicians had pre-university awards (competitions, scholarships, festivals), N = 13 musicians received awards while in university, and N = 22 musicians had professional ensemble placements. The 1000 HGP subset contained N = 210 Males, N = 207 Females, and N = 7 undetermined samples. Since the 1000 HGP subset was a sample of the general population, the HGP subset may include some musicians but we assumed that this was a small percentage. The musician sample and the 1000 HGP subset were in Hardy-Weinberg equilibrium (p = 0.24 for musicians, p = 0.75 for 1000 HGP).

**Table 1:** Demographic variables for the musician sample by carrier status.

Variable	Val/Val (N = 29)	Met-Carriers (N = 21)	Total (N = 50)
Age (at time of data collection)	21.8 ± 3.0	21.7 ± 4.2	21.8 ± 3.5
Age of start for musical training	8.1± 3.1	6.5± 3.9	7.4± 3.7
Sex (M/F)	15/14	8/13	25/25
Handedness (R/L)	29/0	18/3	47/3
Total Years of Training	13.7± 3.0	15.3± 4.1	14.3± 3.6
Years of Training on Primary Instrument	10.9± 3.9	12.0± 5.4	11.7± 4.7
Number of musical achievements	3.1± 1.1	3± 1.3	3.06± 1.2

**Fig 1:**
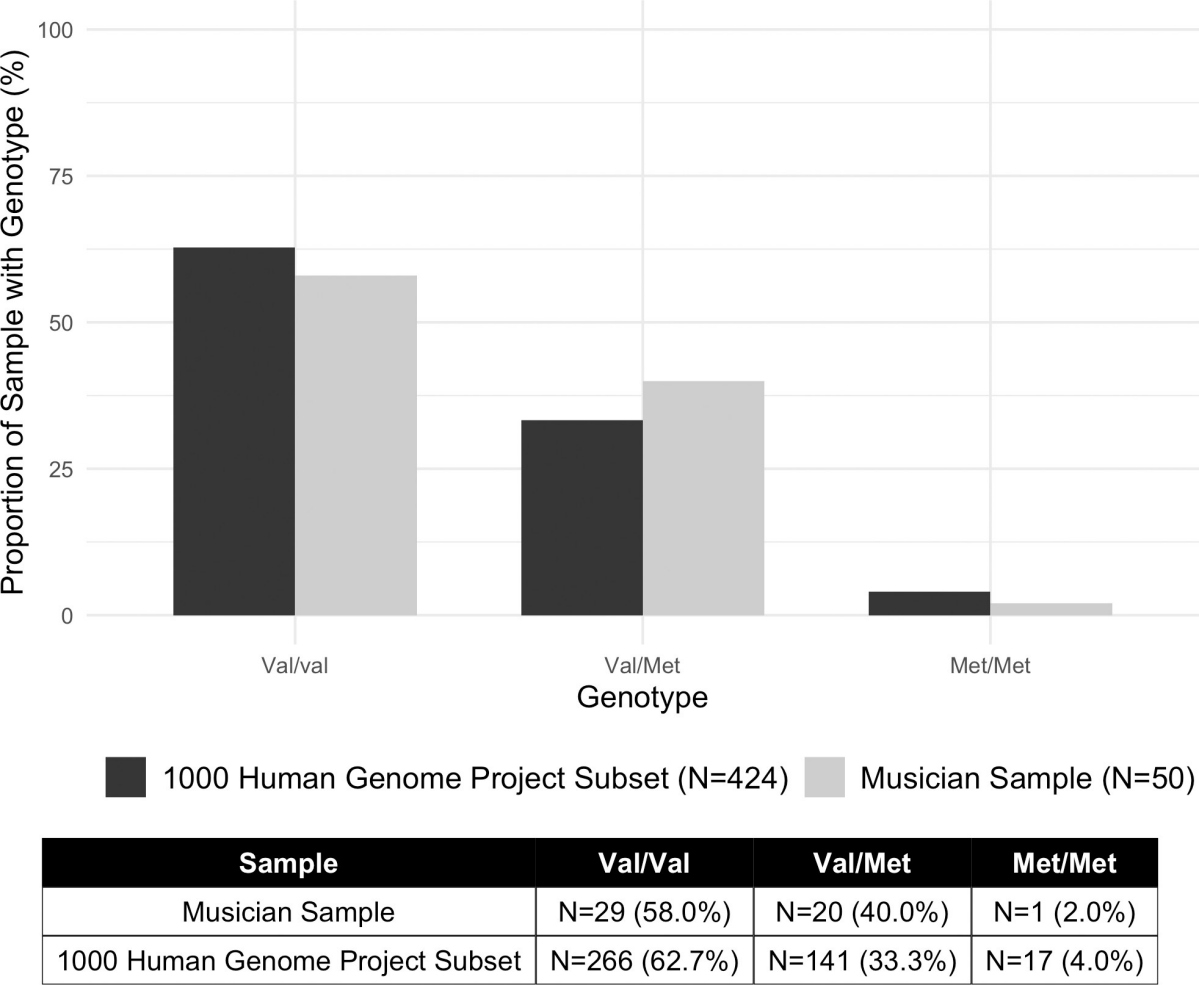
Genotype frequency distributions in the musician sample and 1000 human genome project subset.

**Fig 2:**
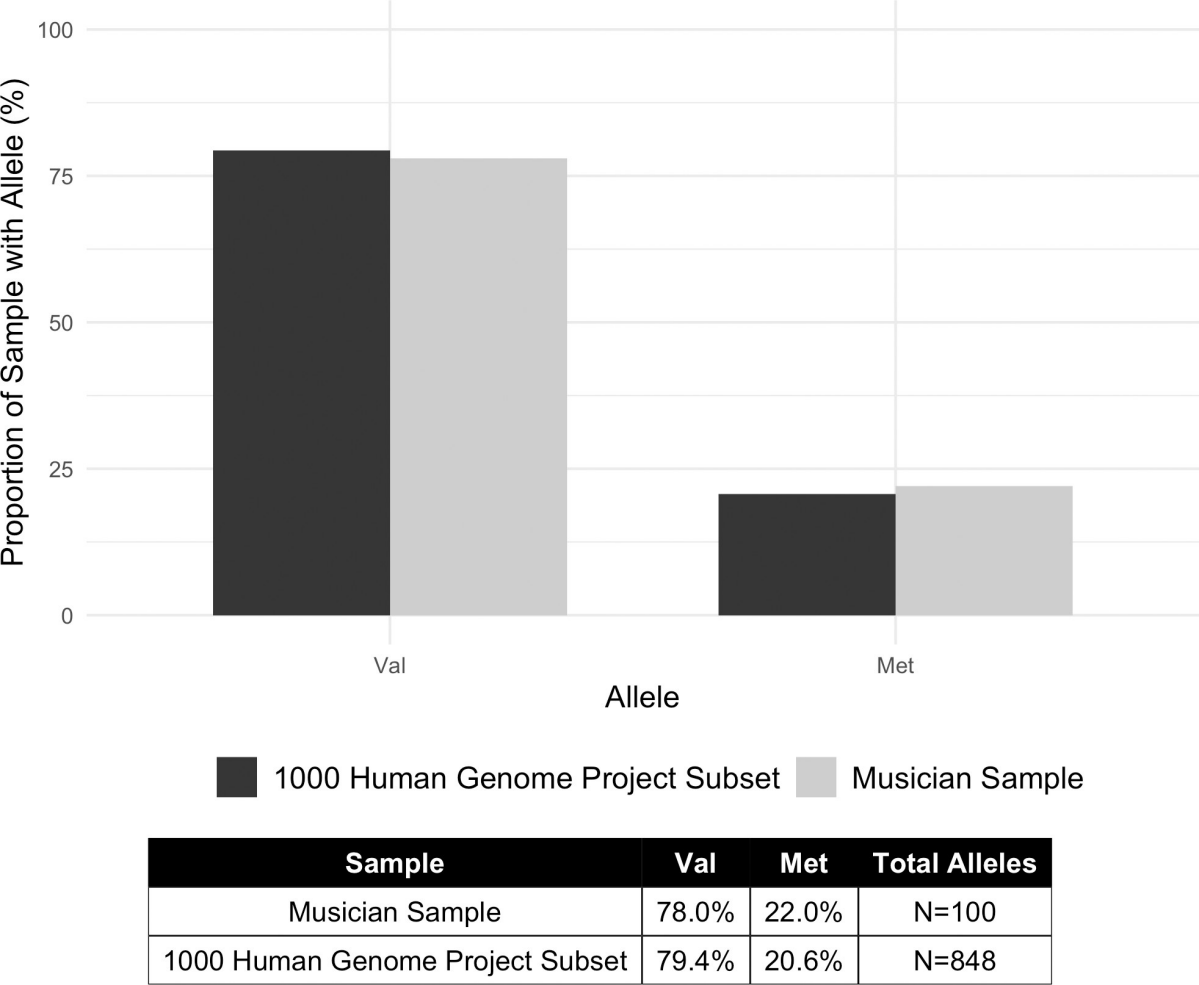
Allele frequency distributions in the musician sample and 1000 human genome project subset.

The results revealed that there were no significant differences in genotype frequencies (p = 0.6447) and allele frequencies (p = 0.8513) (Figs 1 and 2). There were no significant differences between Val/Val and Met-carriers for age (t = 0.074043, df = 34.076, p-value = 0.9414), total years of music training (t = -1.5248, df = 34.546, p-value = 0.1364), years of training on primary instrument (t = -1.4926, df = 34.623, p-value = 0.1446), and age of start of music training (t = -1.5945, df = 39.881, p-value = 0.1187). The number of early starters (before 6.5 years old) versus late starters (after 6.5 years old) were not significantly different (X-squared = 3.6872, df = 1, p-value = 0.05483).

## Discussion

Long-term and intensive music training induces structural and functional brain changes, and enhances short-term plasticity [1, 2, 33, 34]. The presence of the Val66Met BDNF polymorphism (Val/Met or Met/Met) genotype is associated with altered cortical plasticity [5] and deficits in motor learning [6]. The objective of this study was to assess the prevalence of the Val66Met polymorphism in musicians compared to the general population. We report that they were similar. In our pilot study, presence of the Val66Met polymorphism did not overtly limit musicianship. Met-carriers on average started music training early in life at 6.5± 3.9 years old. Therefore, it is possible that intense training beginning early in life and involving long-term deliberate practice [1] required for successful musicianship overcomes inherent MET-dependent deficits in the response to motor training. There is indeed significant evidence that extensive musician training starting early in life influences the motor system function [19, 35]. For example, in a longitudinal training study (24-weeks), Orff-based music training was compared to a sports program and to no training. At post-test and 4-months, children in the music group outperformed control conditions on the Fine motor abilities assessed by the Purdue pegboard test (eye-hand coordination, motor speed, and bimanual coordination) [36]. Musicially-trained early adolescents (typical development) with more than six years of piano showed enhanced fine motor funciton and significangly better elbow and wrist proprioception abilities when compared to children with no musical experience [37]. Children who participated in two years of piano lessons compared to control group (no formal muic instruction) saw significantly greater improvement than controls on the speed subtest and overall score on the Bruinsky-Oseretsky Motor Proficiency [38].

The view of potentially compensatory mechanisms for motor abilities due to long-term training may be consistent with the finding that practice improves Met-dependent deficits in non-musicians [10]. A meta-analysis of N = 55 studies revealed that single sessions of acute aerobic exercise increase activity-dependent BDNF which may have enhancing effects for motor learning [39]. Thus, music training, an intense motor activity, may also increase activity-dependent BDNF. Differences in brain connectivity between musicians and controls that correlate with years of practice [40] could represent one neural substrate supporting this possibility.

Alternatively, it is possible that Met-dependent deficits alone are mild and not enough to elicit training-dependent deficits in musicians, requiring for example other genetic abnormalities to express [3]. Although the genetics of musical motor timing have been explored [41], the genetics of musical motor learning are not known. Future studies, involving larger “n”s and possibly other plasticity probes could inform on the impact of the Met-Met anomaly, present in only one musician in our sample, on musicianship.

Finally, there may be compensatory mechanisms in music-based motor training that are intrinsic to the perceptual-motor structure in music performance. The auditory system is extremely sensitive to rapid and accurate processing of temporal information [42, 43]. Furthermore, there is strong evidence for auditory-motor coupling in motor control driven by rhythmic stimuli [44–46] and motor performance on musical instruments is guided by making movement instantaneously audible to the performer [47]. Thus, music may create a uniquely augmented feedback/feedforward loop for enhanced motor learning, training, and performing [48–50].

The data in our pilot study may therefore also point to mechanisms in music that may hold importance for recovery and re-learning of motor functions. For example, in neurorehabilitation, the Val66Met polymorphism disrupts motor plasticity in stroke patients and may hinder motor function recovery [9]. However, music-based interventions such as Neurologic Music Therapy [NMT] for stroke patients have shown to induce cortical changes in the organization of the sensorimotor cortex and improve motor function [51, 52]. Therefore, future clinical trials should consider the Val66Met polymorphism status as a predictive variable, which may bring insight into the role of BDNF-dependent plasticity in music-based interventions.
